# *In vitro* antibiofilm efficacy of ertapenem, tobramycin, and moxifloxacin against biofilms grown in a glass bead or CDC Biofilm Reactor®

**DOI:** 10.1371/journal.pone.0318487

**Published:** 2025-02-10

**Authors:** Annika L. Gilmore, Helena Vu, Travis Martinez, Lousili Peniata, Brooke Kawaguchi, David A. Armbruster, Nicholas N. Ashton, Dustin L. Williams

**Affiliations:** 1 Department of Biomedical Engineering, University of Utah, Salt Lake City, Utah, United States of America; 2 Department of Orthopaedics, University of Utah, Salt Lake City, Utah, United States of America; 3 Bone and Biofilm Research Lab, University of Utah, Salt Lake City, Utah, United States of America; 4 Depuy Synthes, West Chester, Pennsylvania, United States of America; 5 Department of Pathology, University of Utah, Salt Lake City, Utah, United States of America; 6 Department of Physical Medicine and Rehabilitation, Uniformed Services University of the Health Sciences, Bethesda, Maryland, United States of America; Laurentian University, CANADA

## Abstract

Laboratory grown biofilms are used to simulate bacterial growth in diverse environmental conditions and screen the effectiveness of anti-biofilm therapies. Recently, we developed a glass bead biofilm reactor that utilizes low broth volume to provide high-throughput biofilm growth for testing and translation across the research continuum (e.g., benchtop assays to preclinical models). Bioburden per mm^2^ surface area of *Staphylococcus aureus* and *Pseudomonas aeruginosa* biofilms were comparable on beads and CDC Biofilm Reactor® coupons. In this study, we hypothesized that biofilms grown on beads would be more susceptible to ertapenem, moxifloxacin, and tobramycin than those grown on coupons. Results indicated a significant reduction in *S. aureus* bioburden on glass beads compared to glass coupons following treatment with ertapenem (*p* = 0.005) and tobramycin (*p* = 0.014). *P. aeruginosa* biofilms had smaller differences in antibiotic response between the two systems. There was a significantly greater reduction in bead *P. aeruginosa* biofilm than coupon when treated with tobramycin (*p* = 0.035). This work offered insight into how the bead biofilm reactor could be used as a tool for antibiotic screening and translation across the continuum of *in vitro* to *in vivo* systems that support development of antimicrobial technology.

## Introduction

Researchers often use laboratory grown biofilms to predict the antimicrobial and clinical potential of anti-biofilm therapies [1,2]. There are myriad biofilm reactor systems to accomplish this; each provides unique properties to replicate biofilms in different environments. There are two broad categories of biofilm growth: static and dynamic. Static growth often occurs in closed, “batch” styled reactor environments that lack continuous replacement of nutrients. For example, the minimum biofilm eradication concentration (MBEC) titer plate (previously Calgary biofilm device) is a commonly used static system to determine the amount of antibiotic required to reduce bioburden of single-layered biofilms grown on pegs [3,4]. Dynamic, comparatively, has continuous nutrient replacement and often incorporates high shear via spinning or stirring the growth medium. The most common and widely validated dynamic growth system is the CDC Biofilm Reactor® in which biofilms can be grown on 24 coupons in a turbulent, annular-like reactor under batch and flow phase.

The CDC Biofilm Reactor® is widely published on, yet the system is limited by substrate number and bulk [5,6]. Translating the biofilm-ridden substrates from *in vitro* to *in vivo* applications is challenging [7,8]. We recently developed a bead biofilm reactor containing a custom stainless-steel insert with divots designed to hold fifty roughened bead substrates, allowing them to be exposed to low (40 rpm) shear force for biofilm growth [9]. Glass beads were selected to mimic environmental debris such as soil that often contaminates open wound sites following traumatic injury. The beads can be used for high-throughput *in vitro* screening or as *in vivo* inocula [10]. *Staphylococcus aureus* and *Pseudomonas aeruginosa* biofilms grown on glass beads develop comparable bioburden (colony forming units (CFUs)) per surface area [9]. However, we did not previously determine the antibiotic susceptibility of biofilms grown on glass beads, nor compare outcomes to biofilms grown in a standardized CDC Biofilm Reactor®. We hypothesized that, based on nuance differences in growth parameters, biofilms grown on glass beads would be less robust and thus more susceptible to antibiotics than biofilms grown on glass coupons in a CDC Biofilm Reactor®.

Three antibiotics were selected for comparison: ertapenem, moxifloxacin, and tobramycin. These are military-relevant and apply to our broader orthopedic research program. Ertapenem and moxifloxacin are broad-spectrum antibiotics administered quickly after US soldiers sustain front-line injuries [11]. Clinicians commonly deliver tobramycin on-label (i.e., intravenously (IV) or intramuscularly (IM)) to manage Gram-negative-related infections [12], or off-label as a local antibiotic powder sprinkled into wounds [13].

Ertapenem is a beta-lactam that inhibits bacterial cell wall synthesis. Adding a carbapenem structure to the beta-lactam ring enhances its resistance to bacterial-produced beta-lactamases. Carbapenems, including ertapenem, are generally safe and well-tolerated. While ertapenem works effectively against a wide range of bacteria, certain Gram-negative pathogens like *Klebsiella*, *Pseudomonas*, and *Acinetobacter* often exhibit resistance. Further, sub-inhibitory concentrations of carbapenems may induce phenotypic changes in bacteria that promote biofilm formation [14]. Despite these limitations, civilians commonly receive carbapenems as prophylaxis following traumatic injury and ertapenem remains an antibiotic of choice amongst frontline US military personnel.

Moxifloxacin targets bacterial DNA synthesis, hindering both survival and reproduction of several anaerobic and mixed bacterial populations [15]. However, the risk-benefit profile of moxifloxacin must be carefully evaluated before use as serious adverse side effects such as tendinosis may occur in certain patient populations [16]. Moxifloxacin is the prophylactic antibiotic of choice for US soldiers to self-administer immediately following traumatic injury.

Tobramycin, an aminoglycoside, is commonly administered to treat serious infections and is rapidly absorbed by surrounding muscular tissue following administration [17]. By binding the 16S rRNA of the bacterial 30S ribosomal subunit, tobramycin inhibits protein synthesis and causes the accumulation of misfolded proteins to develop within the bacterial cell. Other researchers have found aminoglycosides to be primarily effective against metabolically active cells [18,19]. Nevertheless, Tobramycin is commonly used to treat orthopedic infections as it is generally well tolerated and effective against a broad spectrum of pathogens.

Determining the susceptibility profiles of biofilms against antibiotics like these can directly influence clinical decisions. Up to 75% of infections are biofilm related [20]. Such infections are often recalcitrant and require extensive regimens to remove bioburden [21]. Nutrient and/or oxygen gradients within a biofilm may influence persister or dormant cell populations at the core, which can directly affect susceptibility to antibiotics [22,23]. Notably, phenotypic characteristics of biofilms, such as antibiotic tolerance, can differ depending on the environment and available nutrients; *S. aureus* may form large multicellular plumes when cultured in a CDC biofilm reactor, or smaller aggregates when imaged *in vivo*.

Screening anti-infective therapies against biofilms grown in reactor systems can elucidate biofilm-specific characteristics and antibiotic responses. Biofilm susceptibility profiles are highly distinct from planktonic outcomes. While individual planktonic cells are susceptible to antibiotic therapy, biofilm communities of the same species often exhibit increased tolerance [23,24]. Likewise, biofilm antibiotic tolerance can differ from structure to structure, depending on the architecture, age, and strain [23]. Biofilm extracellular polymeric substances (EPS) can hinder antimicrobial penetration, enzymes can inactivate antibiotics, and cellular metabolic differences may reduce antibiotic uptake [25,26]. As new anti-biofilms technologies are developed, most require *in vitro* screening. Therefore, a unique biofilm reactor system that can be translated through the continuum of *in vitro* and *in vivo* testing may influence clinical decision making and offer another growth system more representative of biofilm wound contaminant.

## Methods

### Reagents and materials

Brain heart infusion (BHI) broth was sourced from Becton, Dickinson and Company (Franklin Lakes, NJ), cation-adjusted Mueller Hinton broth (CAMHB) from Hardy Diagnostics (Santa Maria, CA), and 10x phosphate buffered saline (PBS) from Thomas Scientific (Swedesboro, NJ). Ten x PBS was diluted to 1x with deionized (DI) water before being autoclaved. Tryptic soy agar (TSA) plates were prepared with a base from MilliporeSigma (Burlington, MA).

Different classes of antibiotics were selected for military and orthopedic relevance [11,27–29]. Ertapenem sodium was sourced from Research Products International (Mount Prospect, IL), moxifloxacin hydrochloride from Chem-Impex (Wood Dale, IL), and tobramycin from Medisca (Irving, TX).

### Bacterial isolates

Isolates were purchased from the American Type Culture Collection (ATCC) and were selected because of their orthopedic infection prevalence [30–32] and biofilm forming potential [33,34]. Frozen stocks of *Staphylococcus aureus* ATCC 6538 and *Pseudomonas aeruginosa* ATCC 27853 were maintained in BHI with 30% glycerol. Isolates were subbed onto TSA plates and incubated at 37°C overnight 1–2 days before use.

### Reactor design and materials

**Fig 1:**
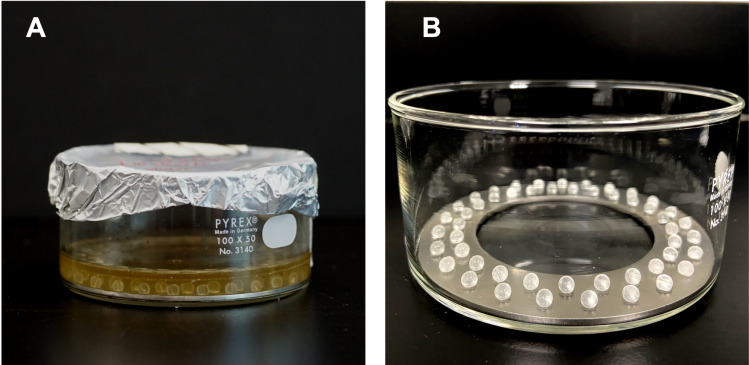
Biofilm bead reactor for low shear, high-throughput growth of biofilms on 50 substrates. (A) *S. aureus* biofilm growth in the bead reactor. (B) Fifty glass beads were stabilized by a 100 mm × 50 mm insert in a petri dish.

The bead reactor [9] (Fig 1A and B) was assembled with a 100 mm x 50 mm Pyrex crystalizing dish (Charleroi, PA), a custom-machined 304 stainless steel ring insert, and fifty 4 mm diameter borosilicate glass beads (Sigma Aldrich; St. Louis, MO).

**Fig 2:**
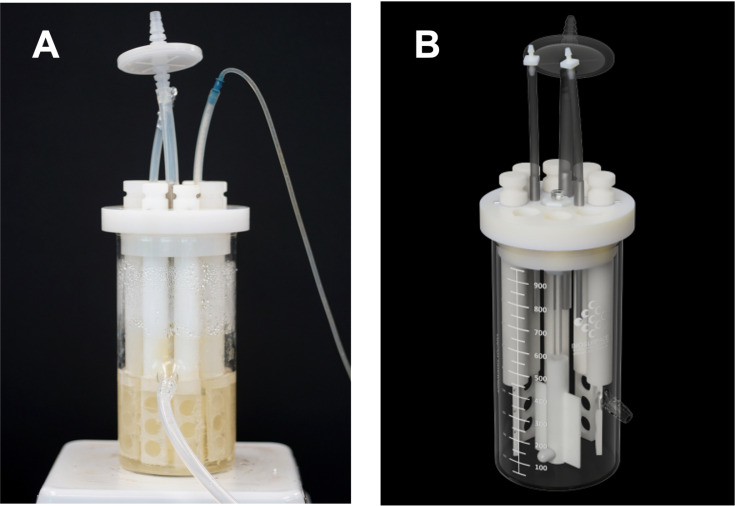
Standardized BioSurface Technologies CDC Biofilm Reactor® for dynamic, high-shear biofilm growth on 24 coupons. (A) *S. aureus* biofilm grown in 100% BHI. (B) Image of empty CDC Biofilm Reactor® reprinted with permission from BioSurface Technologies.

A CDC Biofilm Reactor® (Figs 2A and B) comprised of eight polypropylene reactor arms and 24 borosilicate glass coupons were sourced from BioSurface Technologies (Bozeman, MT). Tubing, barbed connectors, and a peristaltic pump to flow broth into the CDC Biofilm Reactor® were purchased from Cole-Palmer (Vernon Hills, IL).

Both the glass beads and borosilicate coupons were roughened by hand with 60-grit sandpaper (3M; Maplewood, MN). All glass parts were cleaned with Alconox (White Plains, NY) and DI water before autoclaving. Reactor parts were assembled prior to autoclaving. Beads were placed into divots in a biosafety cabinet prior to use.

### Biofilm growth

A 0.5 McFarland standard (~7.5 × 10^7^ colony forming units (CFUs)/mL) of each bacterium was prepared from a fresh culture. One mL of the 0.5 McFarland standard was added to 50 mL of 50% BHI in the bead reactor and 500 mL of 100% BHI in the CDC Biofilm Reactor®. The bead reactor was placed into a shaking incubator at 37° C and 40 rpm for 72 h. Every 24 h, broth was manually exchanged and replaced using a serological pipette in a biosafety cabinet (Fig 1). For the CDC Biofilm Reactor®, batch-phase growth occurred for 24 h on a hot plate at 37° C and baffle rotation of 130 rpm (Fig 2). A 10% BHI solution flowed through the reactor for another 24 h at 6.94 mL/min. The total CDC Biofilm Reactor® growth time was 48 h.

### Scanning electron microscopy imaging

Coupon (n = 2) and bead substrates (n = 8) were removed immediately after reactor growth and fixed with modified Karnovksy’s fixative–2.5% glutaraldehyde and 4% formaldehyde in 0.2 M PBS—at room temperature for 24 h. Fixed substrates were then dehydrated in increasingly concentrated ethanol (EtOH) solutions (70%, 80%, and 90%) for 1–3 h each. The substrates were left in 100% EtOH overnight, then air dried. Substrates were placed onto a scanning electron microscopy (SEM) stage with carbon tape and gold coated with a Hummer 6.2 gold sputter coater (Anatech LTD). Biofilms dwelling on substrates were imaged in secondary electron mode with a JEOL JSM-6610 SEM (Tokyo, Japan).

### Antibiotic treatment

The minimum inhibitory concentration (MIC) of each antibiotic was determined against both bacterial strains using the Clinical & Laboratory Standards Institute protocol [35]. In brief, 50 µL of CAMHB were added to columns 2–12 followed by 50 µL of 256 μg/mL antibiotic in columns 1–2. The solution was mixed with a multichannel pipette, and 50 µL was removed and added to wells in column 3. A 1:2 dilution was continued until column 11 for a final concentration range of 64 to 0.0125 μg/mL. A 0.5 McFarland standard was prepared from a fresh culture of bacteria and diluted 1:100; 50 µL of McFarland standard was added per well (~7.5 × 10^5^ CFU/mL) in columns 2–12. Column 1 was reserved as a negative control and column 12 a positive bacterial control. Plates were incubated at 37°C overnight and MICs were determined the following day.

Ten mg/mL primary stock solutions were prepared in scintillation tubes from weighted antibiotic powder suspended in PBS. Primary stocks were diluted with 50% BHI to obtain 100 µg/mL treatment concentrations. To reflect an appropriate ratio of biofilm surface area to treatment volume (SA/V) between different substrate types (253.4 mm^2^ for CDC Biofilm Reactor® coupon and 50.3 mm^2^ for bead), 1 mL of treatment was used for beads and 5 mL for CDC Biofilm Reactor® coupons. Following preparation of conical treatment tubes (Kimex), biofilm containing coupons and beads (n = 6 per treatment, reactor type, and run) were aseptically removed from reactors and gently placed into capped tubes. Biofilms in treatment were incubated at 37°C and 40 rpm in a shaking incubator for 24 h.

### Microbial quantification

Remaining bacteria was quantified both from treatment broth and on substrates. Treatment tubes were removed from the incubator and 200 µL of sterile glass sand was added to further disrupt adhered biofilm during a vortex-sonicate-vortex (VSV) protocol (1 min, 10 min, and 30 s). To reduce the effects of residual kill, 1 mL of the resulting solution was pipetted from the treatment tube into a microcentrifuge tube and pelleted by centrifugation for 3 min at 5000 rpm. Nine-hundred µL of supernatant was removed and replaced with 900 µL of fresh PBS. The centrifugation process was repeated three times to achieve sub-MIC concentrations of residual antibiotic. The VSV procedure was repeated with an additional 100 µL of sterile glass sand being added. Samples (n = 6 per group, both reactors) were then diluted 10-fold and plated on TSA plates for incubation overnight at 37°C. Colony forming units (CFUs) were counted the following day.

### Statistical analysis

Three trials against *S. aureus* and *P. aeruginosa* were completed with n = 6 substrates per test group (n = 18 total samples). Bioburden per substrate area was log_10_ transformed. The average log_10_ CFU/mm^2^ of control, untreated substrates was calculated per trial (n = 3) and the remaining bioburden of antibiotic treated samples were subtracted from the average bioburden of the respective trial controls. The average bioburden reduction was then calculated per trial and standard deviation from the entire data set. A Student’s *t*-test using the average values of bioburden reduction of each trial was used to compare bioburden between CDC Biofilm Reactor® and bead reactor controls, and each treatment.

## Results

### Biofilm SEM imaging

**Fig 3:**
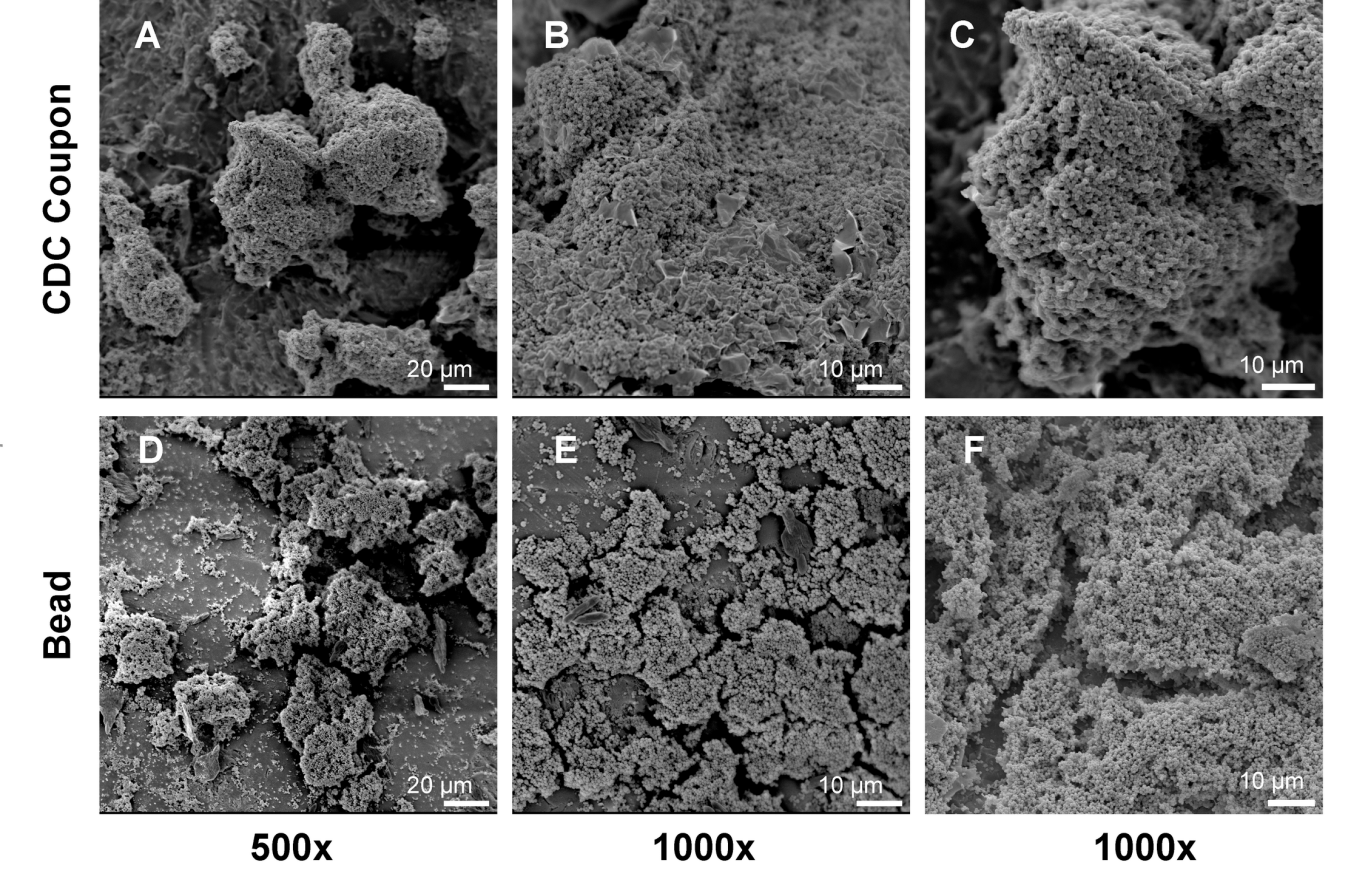
Representative images of *S. aureus* biofilm grown on CDC Biofilm Reactor® coupons and 4 mm diameter glass beads. (A) 500x image of biofilm on a coupon. (B, C) at 1000x, coupon biofilm exhibited thick, multilayered plumes. (D) 500x image of bead biofilm. (E, F) 1000x images of bead biofilm also displayed multiple plumes that appeared slightly less dense than biofilm grown on coupons.

**Fig 4:**
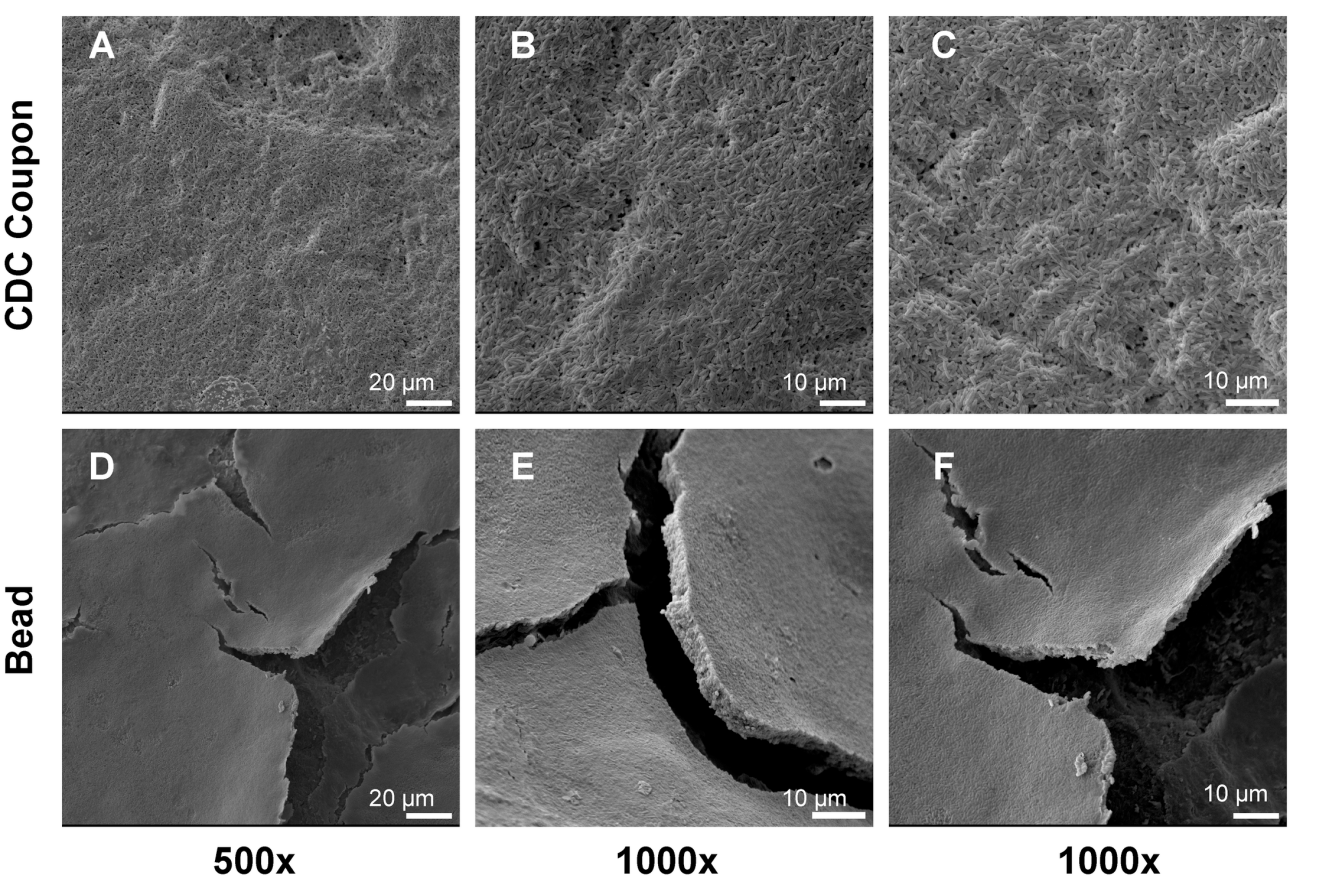
Representative images of *P. aeruginosa* biofilm grown on glass coupons and beads. (A) 500x image of CDC Biofilm Reactor® coupon that, like (B and C) 1000x images, revealed even sheets of *P. aeruginosa*. (D) 500x image of bead biofilm with cracking due to fixation of biofilm onto rounded glass surfaces. (E,F) 1000x images of bead biofilm confirm multilayered sheets of *P. aeruginosa* growth.

SEM images of biofilms grown on coupons or beads revealed consistent, mature structures across each trial. However, the plumes of *S. aureus* grown in the CDC Biofilm Reactor® (Figs 3A–C) looked slightly thicker than those grown in the bead reactor (Figs 3D–F). On both substrate types, *S. aureus* biofilm exhibited larger 3-dimensional plumes around the roughened areas. Even, multilayered sheets of *P. aeruginosa* were visible in SEM images of both CDC Biofilm Reactor® coupons and beads (Fig 4). The overall thickness of coupon biofilm was not immediately evident (Figs 4A–C). Notably, flaking of *P. aeruginosa* biofilm following fixation and dehydration exposed sheet thickness of rounded glass beads (Figs 4D and E). Based on SEM images, we estimated bead biofilm thickness to be approximately 5 bacterial cells.

### Microbial quantification

**Fig 5:**
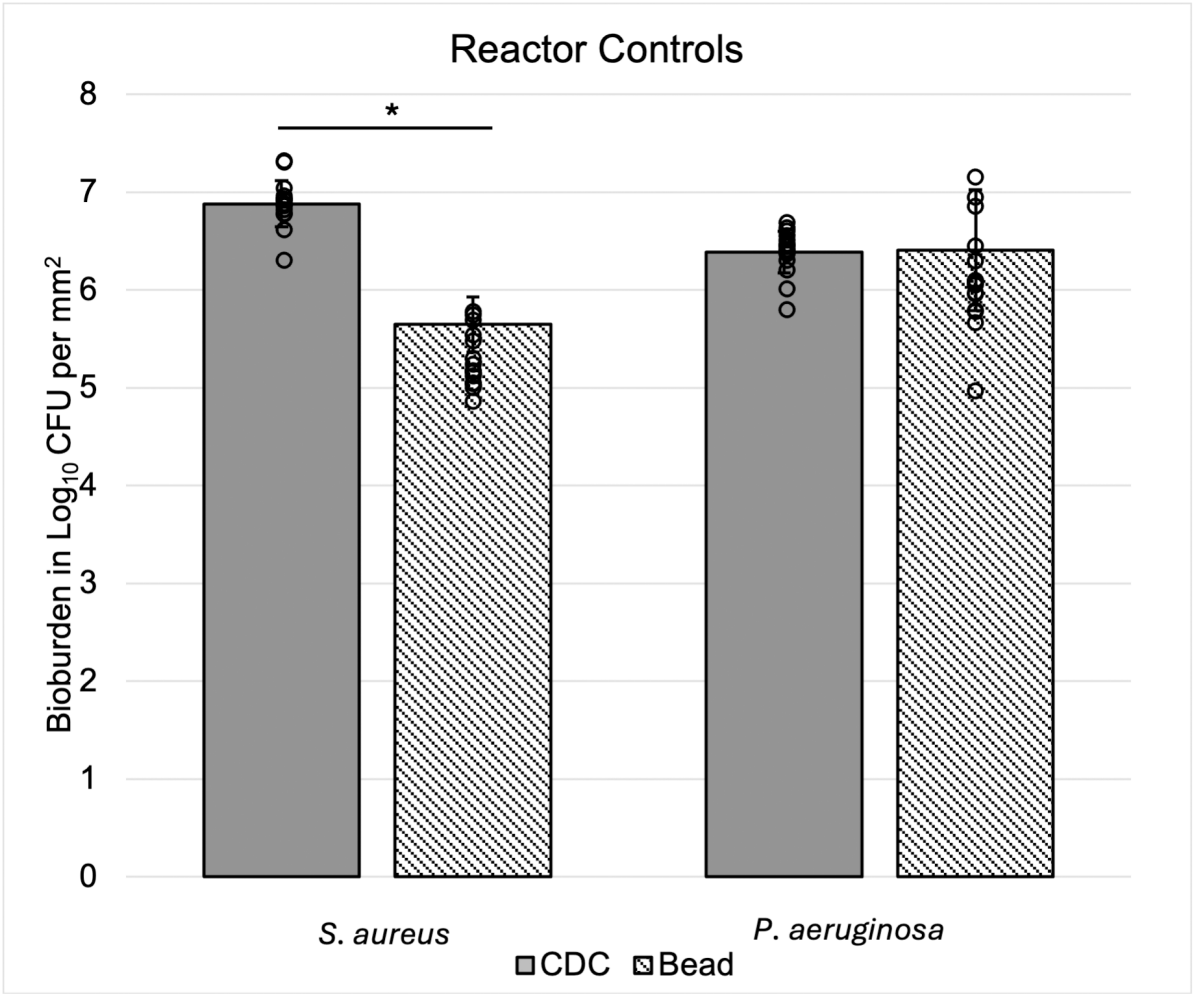
Bioburden in log10 CFU per mm^2^ growth surface area of *S. aureus* and *P. aeruginosa* biofilm. The average (n = 18 bead, n = 17 coupon) of substrates from 3 trials are represented by grey and striped bars. Each black dot denotes an individual quantified substrate. There was a significant difference between coupon and bead *S. aureus* biofilm growth (*p* = 0.003). This was not the case for *P. aeruginosa*.

CFU data can be accessed in the supplemental file ‘S1 CFU data.’ Quantification of substrate bioburden revealed similar *S. aureus* and *P. aeruginosa* CFU per mm^2^ on coupons and beads (Fig 5). CDC Biofilm Reactor® coupons had approximately 1 log_10_ CFU/mm^2^ more *S. aureus* bioburden than beads with an average of 6.88 ± 0.23 log_10_ CFU/mm^2^ compared to 5.65 ± 0.28 log_10_ CFU/mm^2^ per bead. There was not a statistically significant difference between *P. aeruginosa* bioburden between coupons (with an average of 6.39 ± 0.21 log_10_ CFU/mm^2^) or bead (6.41 ± 0.62 log_10_ CFU/mm^2^). Both reactors demonstrated repeatable, reliable *S. aureus* and *P. aeruginosa* biofilm growth.

### Treatment outcomes

*S. aureus* and *P. aeruginosa* were both susceptible (as determined by MIC) to all three antibiotics tested (Table 1). The highest MIC was that of ertapenem against *P. aeruginosa*, which was anticipated based on other studies and its known poor efficacy against that organism. All other MIC values indicated planktonic susceptibility of both organisms.

**Table 1:** MIC of ertapenem, moxifloxacin, and tobramycin against *S. aureus* and *P. aeruginosa.*

Organism	Ertapenem (µg/mL)	Moxifloxacin (µg/mL)	Tobramycin (µg/mL)
*S. aureus* ATCC 6538	0.125	0.03125	0.5
*P. aeruginosa* ATCC 27853	16	0.5	0.5

**Fig 6:**
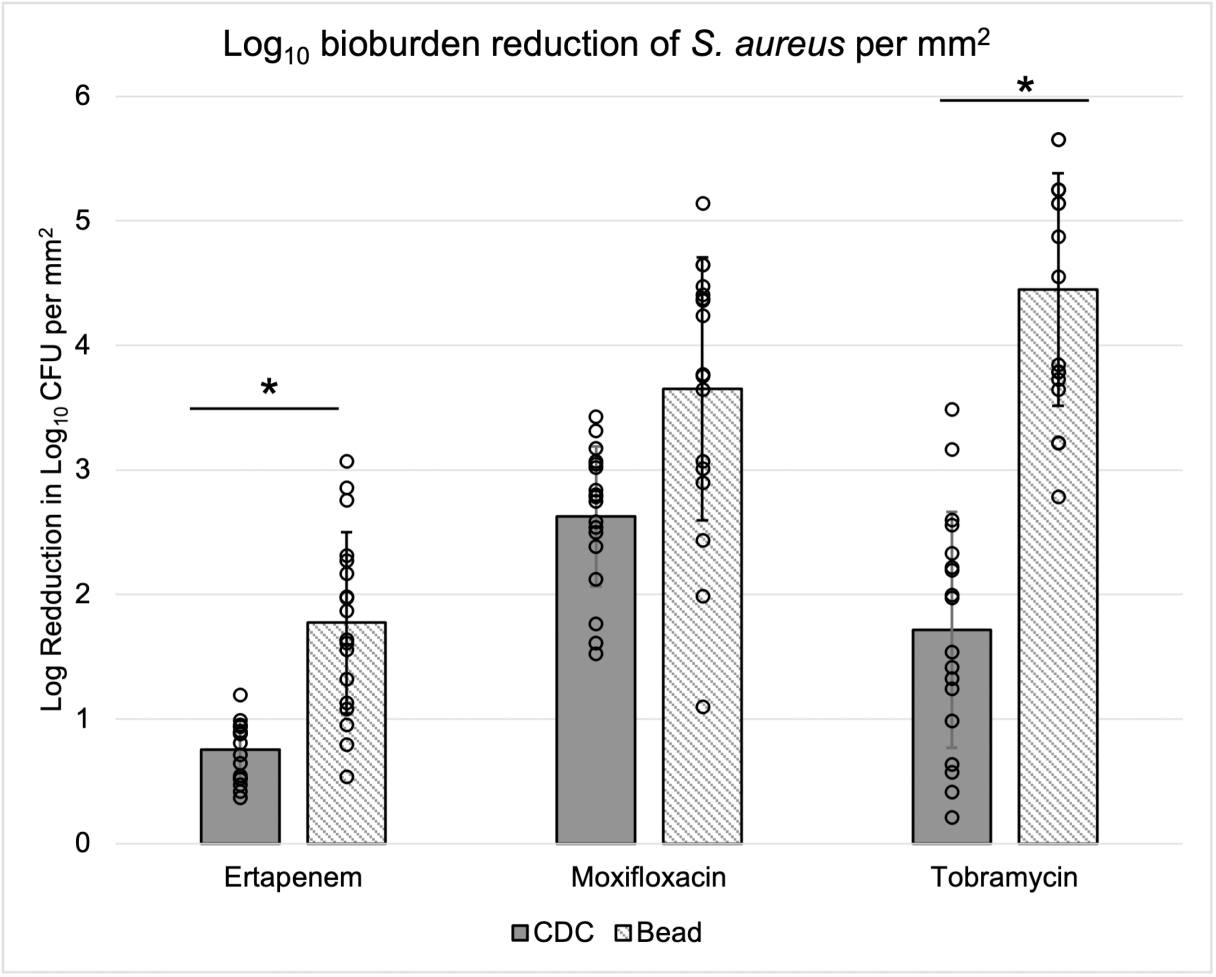
*S. aureus* bioburden reduction calculated in log_10_ CFU per mm^2^ growth surface area. Bead and coupon biofilms were treated for 24 h of in 100 µg/mL antibiotic suspended in 50% BHI broth. Each bar represents the average bioburden of 18 samples. Solid grey represents the CDC Biofilm Reactor® coupons and diagonal lines the bead reactor. Black dots denote the specific CFU of each bead quantified (n = 18 per bar). There was a significant difference (*p* < 0.05) between the average bioburden of CDC and bead biofilm treated with ertapenem (*p* = 0.005) and tobramycin (*p* = 0.014).

*S. aureus* bacteria survived on substrates and in treatment suspensions following antibiotic treatment at concentrations greater than 100x the determined MIC (Fig 6). Seven (of 18) *S. aureus* biofilm beads were eradicated beyond detectable levels by tobramycin. For both *S. aureus* and *P. aeruginosa*, tobramycin and moxifloxacin achieved greater reduction in bioburden than ertapenem. The average reduction in *S. aureus* bead biofilm burden was 1.77 ± 0.72 log_10_ CFU/mm^2^ for ertapenem, 3.65 ± 1.06 log_10_ CFU/mm^2^ for moxifloxacin, and 4.56 ± 1.06 log_10_ CFU/mm^2^ for tobramycin. CDC-grown *S. aureus* biofilm had lower reductions in bioburden with an average of 0.76 ± 0.23 log_10_ CFU/mm^2^ for ertapenem, 2.63 ± 0.56 log_10_ CFU/mm^2^ for moxifloxacin, and 1.72 ± 0.95 log_10_ CFU/mm^2^ for tobramycin.

**Fig 7:**
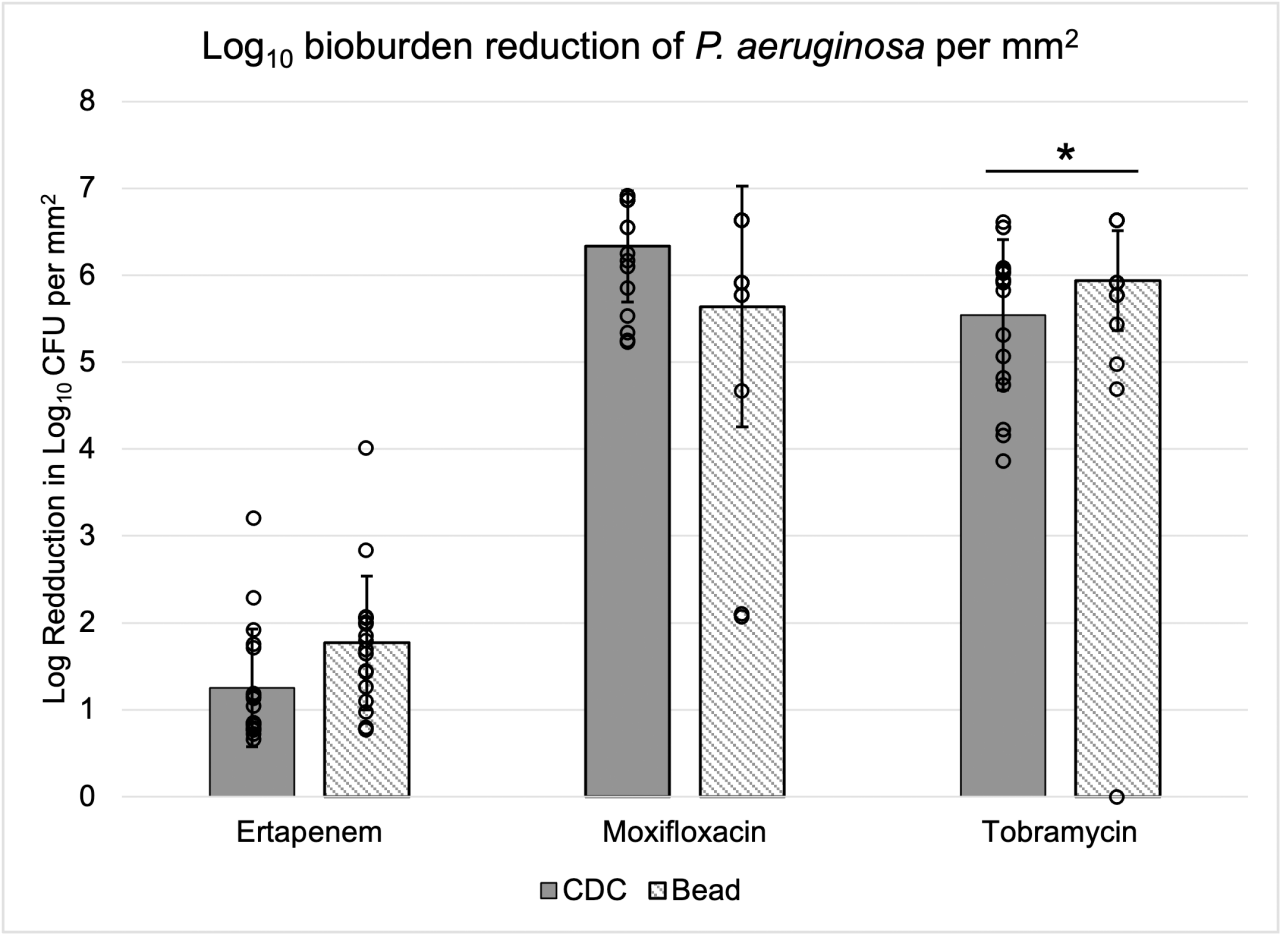
*P. aeruginosa* bioburden reduction in log_10_ CFU per mm^2^. Biofilms were treated for 24 h in 100 µg/mL antibiotic suspended in 50% BHI broth. Solid grey bars represent the average CFU per three trials for CDC Biofilm Reactor® coupons and diagonal lines the beads. Black dots denote the specific CFU of each bead quantified (n = 6 per trial). There was a significant difference (<0.05) between average bioburden reduction of tobramycin-treated coupons and beads (*p* = 0.035).

Reduction of *P. aeruginosa* coupon and bead bioburden was more comparable than *S. aureus* substrates (Fig 7). This could be a result of similarly thick biofilm structures. Moxifloxacin and tobramycin efficacy against bead *P. aeruginosa* biofilm varied and bioburden was reduced to below detectable levels on some coupons and beads. Of 18 coupons, biofilm of 10 were eradicated beyond detectable levels by moxifloxacin and on 14 (of 18) beads. Tobramycin eradicated biofilm from 2 (of 18) coupons and 13 (of 18) beads. The average reduction in *P. aeruginosa* bead biofilm burden was 1.77 ± 0.77 log_10_ CFU/mm^2^ for ertapenem, 5.85 ± 1.53 log_10_ CFU/mm^2^ for moxifloxacin, and 6.16 ± 0.63 log_10_ CFU/mm^2^ for tobramycin. CDC-grown *P. aeruginosa* biofilm had average reductions of 1.25 ± 0.68 log_10_ CFU/mm^2^ for ertapenem, 6.10 ± 0.46 log_10_ CFU/mm^2^ for moxifloxacin, and 5.50 ± 0.82 log_10_ CFU/mm^2^ for tobramycin. Overall, the degree of reduction appears proportional to the shear in which the biofilm was grown with the high shear CDC Biofilm Reactor® biofilm, generally, being more difficult to eradicate than bead.

## Discussion

Previously, we developed a reactor that consistently produced *S. aureus* and *P. aeruginosa* biofilms on glass bead substrates in a low-shear environment [9]. In the current study, we advanced the development of this reactor by testing the hypothesis that biofilms grown on glass beads would be more susceptible to antibiotics than those grown on glass coupons in a CDC Biofilm Reactor®. The hypothesis was supported with *S. aureus* biofilms, but unsupported with *P. aeruginosa.* Future work will be needed to clarify why these discrepancies occurred, but it may be attributed to nuance differences in growth conditions including shear forces and batch vs. flow phases that may have influenced biofilm densities and accompanying tolerance—factors that have been well-defined previously [23,36,37].

*S. aureus* biofilm density is known to be influenced by shear force and a dynamic nutrient profile, which the CDC Biofilm Reactor® provides [38–40]. Kostenko et al. (2010) found *S. aureus* biofilm morphology to correlate strongly with shear stress, increased biofilm tolerance, and architectural size [41]. In the turbulent environment of the CDC Biofilm Reactor®, it is possible *S. aureus* biofilms develop more compactly than those in the bead reactor system.

Low shear in the glass bead reactor appeared to produce less dense *S. aureus* biofilms than those grown in the high shear CDC Biofilm Reactor®. When viewed by SEM, the general *S. aureus* biofilm plume morphology appeared similar on glass beads and coupons, but there was a noticeable difference in plume height. Only the tops of coupon biofilm easily came into focus (Figs 3A–C), whereas most of the plume structures were visible on beads (Figs D–F). Based on literature, *S. aureus* plume height and density may have contributed to antibiotic susceptibility [38]. This could explain the discrepancy between coupon and bead log reduction following treatment with tobramycin (Fig 6), in particular, though future studies are needed to determine the ratios of bacterial cell populations.

Morphology and density did not appear to differ between *P. aeruginosa* biofilms on beads or coupons. In both cases, sheet-like morphologies were present. Cracking of bead biofilm allowed for visualization of sheet thickness (Figs 4D–F). Future work could aim to image cross sections of biofilm grown on coupons for a direct comparison of sheet thickness. We anticipated *P. aeruginosa* biofilm grown on beads and coupons to be similarly dense because quantification of control substrates indicated similar bioburden per surface area of bead and coupons (Fig 5), which may have correlated to equal antibiotic susceptibility profiles of each antibiotic tested (Fig 7).

Several factors may have contributed to denser *S. aureus* yet not *P. aeruginosa* coupon biofilms. First, the growth conditions varied between the CDC Biofilm Reactor® and bead reactor growth protocols. These differences included growth times (48 vs 72 h total), nutrient availability (continuous vs. batch phase), and applied shear force (130 vs 40 rpm). It is well established that growth protocols can significantly impact biofilm development. Shear force and variation in nutrient type and concentration are known alter *S. aureus* biofilm thickness [38,39,41,42]. Second, maintenance of the incubation temperature varied between systems. Hotplates set to 37°C maintained temperature in the CDC Biofilm Reactor® while a closed 37°C incubator likely keep a more consistent temperature within the bead reactor. Lower temperatures tend to promote biofilm formation [43]. Thus, biofilm growth within CDC Biofilm Reactor® might have been advantaged by partial exposure of the system to a lower ambient room temperature. Third, *S. aureus* cells may have generated a different plume base between substrate geometries. Coupons and beads were roughened with the same sanding protocol, however, fluid dynamics surrounding a flat coupon, and a spherical bead likely differed. Bead curvature might encourage different *S. aureus* biofilm adhesion affecting both colonization rate and overall biofilm density [36]. The CDC Biofilm Reactor® and bead reactor procedures differ significantly, yet both produce comparable bioburden per substrate surface area. Evaluating how stress, temperature, and nutrient availability vary has enhanced our understanding of resulting tolerance.

While MIC assays confirmed *S. aureus* and *P. aeruginosa* susceptibility to ertapenem, moxifloxacin, and tobramycin, biofilms of the same organisms were far less susceptible to antibiotic reduction. The mechanism of action of moxifloxacin is inhibits bacterial DNA replication; tobramycin inhibits bacterial protein translation. These antibiotics likely avoid common biofilm tolerance mechanisms such as creation of efflux pumps. Future studies should compare and aim to quantify cell populations (e.g., metabolically active, or dormant) throughout each biofilm. Dormant cells might not be as susceptible to antibiotic killing. Ertapenem had broad spectrum activity against many Gram-negative pathogens, and was effective against planktonic *P. aeruginosa.* Yet ertapenem achieved less than a 3 log_10_ reduction in both *S. aureus* and *P. aeruginosa* bioburden, and in the case of *P. aeruginosa*, appeared to promote biofilm growth. This finding motivates more challenging biofilm-related research when developing and screening anti-infective technologies.

Future research should consider screening against additional pathogens, including polymicrobial biofilms, and treating with lower concentrations of tobramycin and moxifloxacin. Many substrates had no detectable growth, despite utilization of an antibiotic wash method to mitigate false negative results. Additional concentrations and treatment timepoints could help identify further discrepancies between bead and coupon biofilm.

## Conclusions

The bead and CDC Biofilm Reactor® allow for the consistent growth of *S. aureus* and *P. aeruginosa* biofilm advantageous to the testing and preclinical development of anti-biofilm products. This work offers insight into the antibiotic tolerance of bead-grown biofilm, further defining characteristics of this relatively new system in the context of a standardized method. Future work and expanded testing will be needed to validate the system but the data shared suggest the bead reactor may be a worthwhile tool for antibiotic screening and translational research applications.

## Supporting information

S1 Data Original CFU counts per sample. (XLSX)
